# Warming Amplification of Minimum and Maximum Temperatures over High-Elevation Regions across the Globe

**DOI:** 10.1371/journal.pone.0140213

**Published:** 2015-10-13

**Authors:** Xiaohui Fan, Qixiang Wang, Mengben Wang, Claudia Villarroel Jiménez

**Affiliations:** 1 Institute of Loess Plateau, Shanxi University, Taiyuan, Shanxi Province, China; 2 Dirección Meteorológica de Chile, Santiago, Chile; University of Vigo, SPAIN

## Abstract

An analysis of the annual mean temperature (T_MEAN_) (1961–2010) has revealed that warming amplification (altitudinal amplification and regional amplification) is a common feature of major high-elevation regions across the globe against the background of global warming since the mid-20th century. In this study, the authors further examine whether this holds for annual mean minimum temperature (T_MIN_) and annual mean maximum temperature (T_MAX_) (1961–2010) on a global scale. The extraction method of warming component of altitude, and the paired region comparison method were used in this study. Results show that a significant altitudinal amplification trend in T_MIN_ (T_MAX_) is detected in all (four) of the six high-elevation regions tested, and the average magnitude of altitudinal amplification trend for T_MIN_ (T_MAX_) [0.306±0.086 °C km^-1^(0.154±0.213 °C km^-1^)] is substantially larger (smaller) than T_MEAN_ (0.230±0.073 °C km^-1^) during the period 1961–2010. For the five paired high- and low-elevation regions available, regional amplification is detected in the four high-elevation regions for T_MIN_ and T_MAX_ (respectively or as a whole). Qualitatively, highly (largely) consistent results are observed for T_MIN_ (T_MAX_) compared with those for T_MEAN_.

## Introduction

Two key questions related to climate changes in high-elevation regions are whether elevation-dependent warming commonly occurs in these regions, and whether high-elevation regions are warming faster than their low-elevation counterparts [1–5]. During the recent decades, numerous studies have focused on the first issue based on surface observations. Although most studies focused on the mean temperature [6–11], a few other studies also analyzed the minimum temperature and maximum temperature [11–15]. However, the elevation dependency was statistically confirmed only for the minimum temperature anomalies (1979–1993) in the Swiss Alps[12]. On the second issue, Falvey and Garreaud [16] used daily mean temperature series (1979–2006) and identified a strong contrast between surface cooling at low-lying (coastal) stations and warming in the Andes in central and northern Chile. Additionally, greater warming was observed at high-elevation sites than at low-lying sites in the Swiss Alps based on the trends in maximum and minimum temperatures [17–18].

In the latest study, based on a dataset of annual mean temperature (T_MEAN_) series (1961–2010) from 2367 stations around the world, Wang *et al*. [19] revealed that warming amplification (altitudinal amplification and regional amplification) is a common feature of major high-elevation regions across the globe against the background of global warming since the mid-20th century. These authors reached this conclusion by developing the altitudinal warming component extraction equation (AWCE equation), and employing the paired region comparison method. In this study, we further examine whether this holds for annual mean minimum temperature (T_MIN_) and annual mean maximum temperature (T_MAX_).

## Materials and Methods

### 2.1 Data

**Fig 1:**
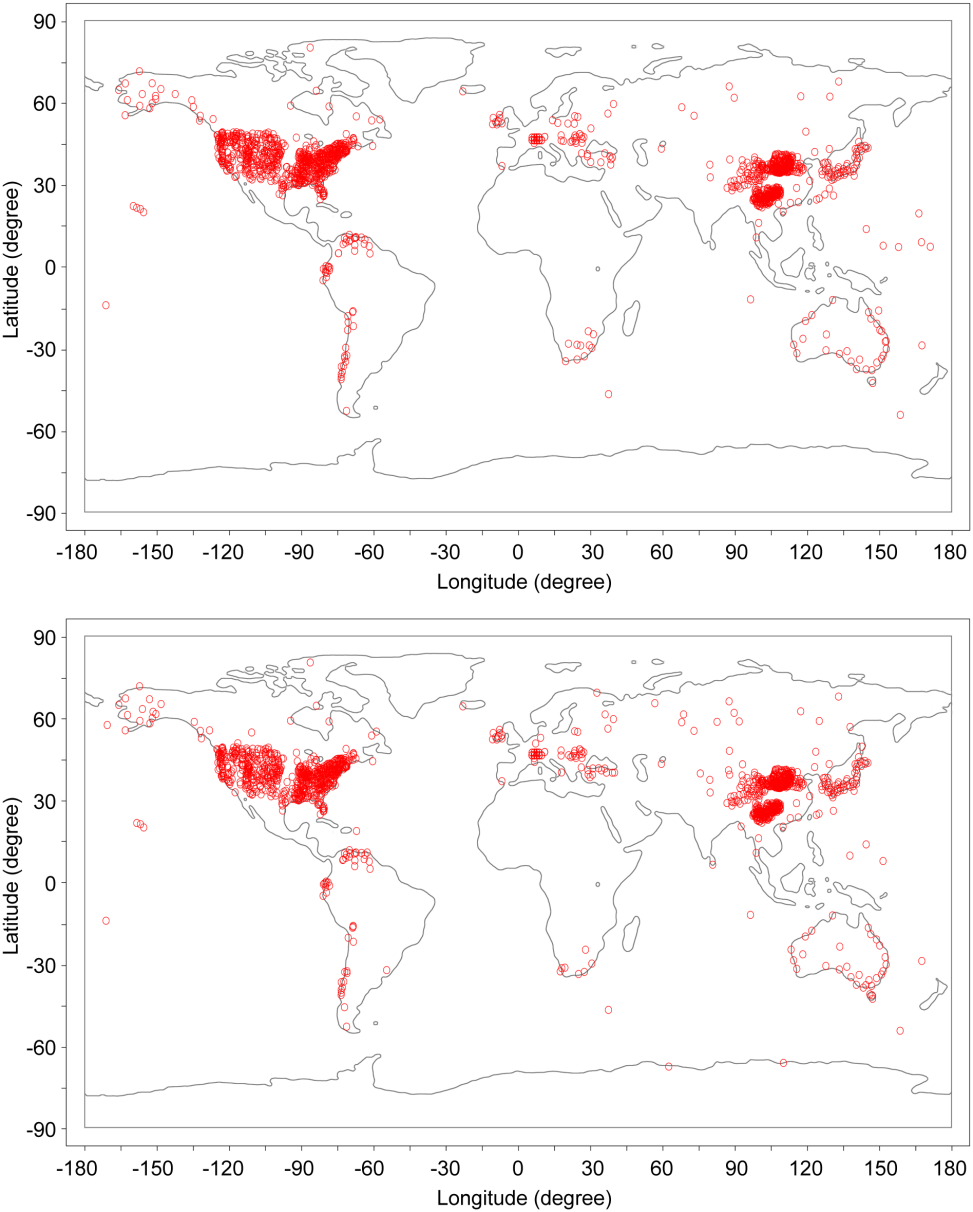
Distribution of 1494 stations with annual mean minimum temperature series (top) and 1448 stations with annual mean maximum temperature series (bottom) used for this study around the globe. The boundaries of continents, generated with the Adobe Photoshop CS6, are not necessarily identical to the original image, and are therefore for illustrative purpose only.

**Fig 2:**
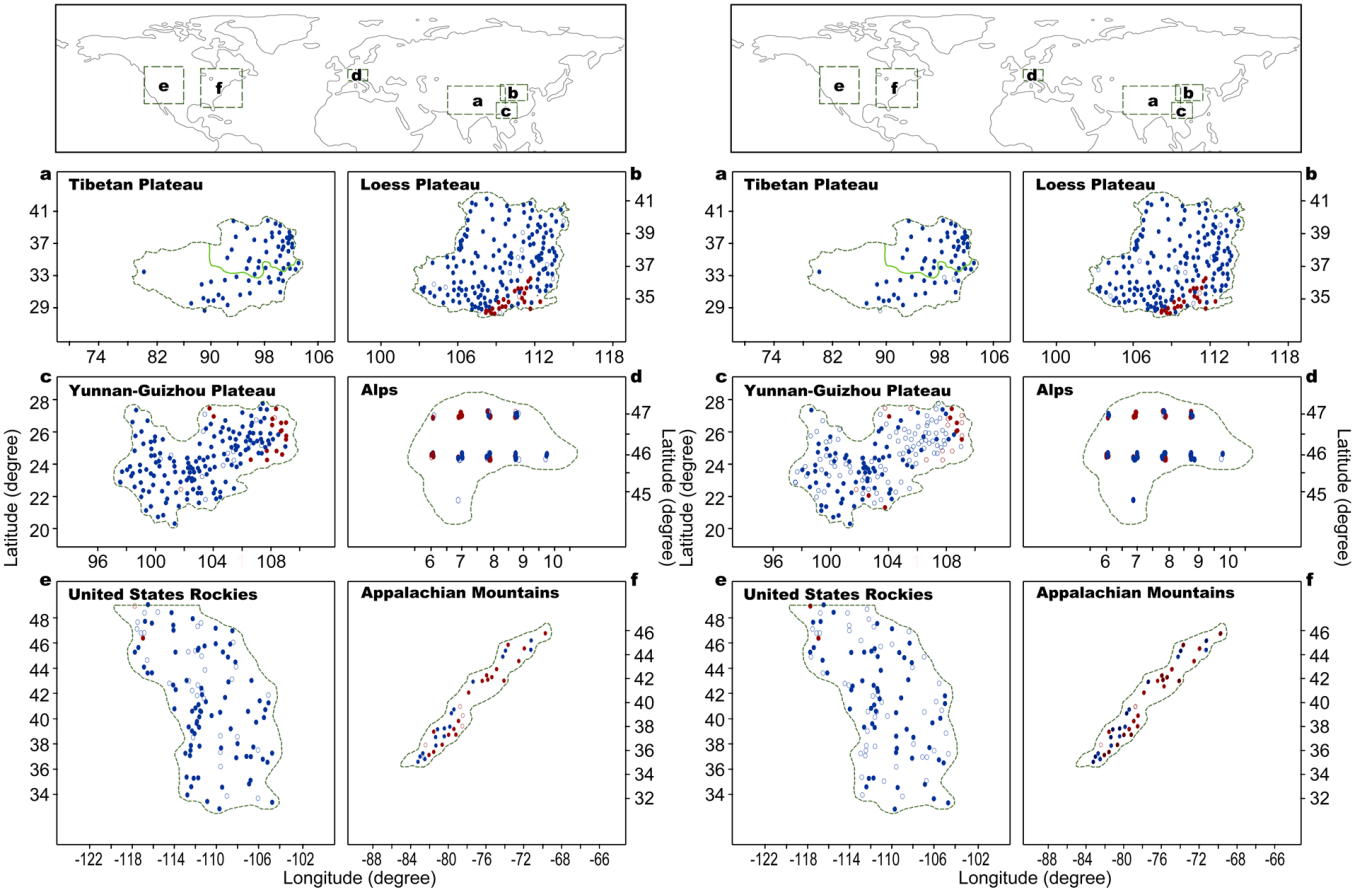
Distribution of stations in the high-elevation regions. The left panels show the stations with annual mean minimum temperature series, and the right panels show the stations with annual mean maximum temperature series. The high-elevation stations at ≥500m asl, and ≥200m to <500 m asl are shown in blue and red colors, respectively. Dots and circles stand for significant and non-significant positive trends, respectively. The boundaries of continents and the high-elevation regions, generated with the Adobe Photoshop CS6, are for illustrative purpose only.

This study used a dataset of 1,494 T_MIN_ and 1,448 T_MAX_ station series (1961–2010) around the world (Fig 1). Of all the stations, 1334 are the same stations, representing 89.3% (92.1%) of the total T_MIN_ (T_MAX_) stations_;_ and 652 (641) T_MIN_ (T_MAX_) stations are sited at the six high-elevation regions tested (Fig 2), of which 636 are the same stations, representing 97.5% (99.2%) of the total high-elevation region T_MIN_ (T_MAX_) stations.

The data were compiled from 6 sources: the Global Historical Climatology Network Monthly (GHCNM) version 3.2.0dataset [20]; the National Meteorological Information Center of China (NMICC); MeteoSwiss; the Latin American Climate Assessment and dataset (LACAD)[21]; the European Climate Assessment & Dataset (ECA&D)[22]; and the National Weather Service of Chile (NWSC). The data for the GHCNM, NMICC, ECA&D and NWSC were available for the entire period 1961–2010, while the data for the LACAD were available for the period 1961–2006.

To obtain the annual time series from the daily minimum and maximum temperatures from the ECA&D and NWSC, two steps (or two criteria) were required. A monthly mean value was first calculated for the available days if no more than 3 days of data were missing in that month; then, an annual mean value was computed from the monthly means if 12 monthly values were present in that year. For the monthly data from other sources, the annual time series was calculated using the second criterion. After the establishment of the T_MIN_ and T_MAX_ series (1961–2010), the series that had at least37yearsof complete data (i.e., 12 months per year) were selected for homogeneity testing using RHtests V3 [23]. The series that had obvious inhomogeneity were excluded. However, the series from the LACAD with at least 30 years of complete data were also included in the homogeneity test because the source dataset ended in 2006, the available stations in the Andes were very sparse, and no other data are available at present. The final dataset consisted of 909 (874), 459 (459), 58 (53), 36 (33),17 (17) and 15 (12)T_MIN_(T_MAX_) stations from the GHCNM; NMICC, MeteoSwiss, LACAD, ECA&D and NWSC, respectively, with 37, 41, 37, 30, 41and 40yearsof complete data, respectively.

### 2.2 Methods

#### 2.2.1 Test of temperature trend

The trend for a station (or a region as a whole) was extracted from the anomalies (relative to the 1961–1990 mean) using the Mann-Kendall method [24–25]. The trend slope was estimated using Sen’s method [26] and the trend significance was determined using the Mann-Kendall test [24–25] with an iterative procedure [27].

#### 2.2.2 Test of altitudinal amplification

The methodology for evaluation of the altitudinal amplification trend for T_MIN_ (T_MAX_) within a high-elevation region was exactly the same as for T_MEAN_ in the previous study [19], consisting of the following four steps:

1. Transformation of altitude (in meter), latitude (in degree) and longitude (in degree) into *ALT*, *LAT* and *LONG* (all in km) for each station using the following equations,
  ALT=altitude/1000, (1)
  LAT=latitude×111.317, (2)
  LONG=longitude×π×R×cos(latitude)/180. (3)
  where 111.317 (expressed in km) is the distance constant per degree of latitude, and *R* is the radius of the Earth. Because the distance between two degrees of longitude changes with latitude, eq (3) is necessary.
2. Estimation of the effect coefficients of altitude, latitude and longitude (*EC*_ALT_, *EC*_LAT_ and *EC*_LONG_, respectively) on a regional scale using stepwise regression. This procedure was performed with the model of fit *y* = b_1_ *x*_1_ + b_2_ *x*_2_ +b_3_ *x*_3_ + *c*, where the long-term (1961–2010) average T_MIN_ and T_MAX_ values (***T***_***AVG***_ in general, °C) and the *ALT*, *LAT* and *LONG* values of the individual stations within a region were taken as the dependent (*y*) and independent variables (*x*_1_, *x*_2_, and *x*_3_), respectively. The negative values of the regression coefficients (b_1_, b_2_ andb_3_) estimated for *ALT*, *LAT* and *LONG* (i.e., the temperature lapse rates along the altitudinal, latitudinal and longitudinal gradients) were taken as *EC*_ALT_, *EC*_LAT_ and *EC*_LONG_, respectively. When an independent variable was not introduced (i.e., the partial correlation coefficient for it was not significant at the 0.05 level), its effect coefficient was considered to be zero.
3. Extraction of the warming component of altitude (*Q*_ALT_) from the station warming rate (*Q*_TOTAL_) for each station within a high-elevation region using the following equation,
  $$Q_{ALT}=\frac{Q_{TOTAL}\times EC_{ALT}\times ALT}{\sqrt{{\left(EC_{ALT}\times ALT\right)}^{2}+{\left(EC_{LAT}\times LAT\right)}^{2}+{\left(EC_{LONG}\times LONG\right)}^{2}}}.$$ (4)
  where *Q*_TOTAL_ in T_MIN_ and T_MAX_ is expressed in °C 50-yrs^-1^, and *ALT*, *LAT* and *LONG* are all expressed in km for each station, and *EC*_ALT_, *EC*_LAT_ and *EC*_LONG_ are constant values for every station within the region, and are expressed in °C km^-1^. The result, *Q*_ALT_, is also expressed in °C 50-yrs^-1^.
4. Test of the altitudinal amplification trend for each region. Based on the *Q*_ALT_ values extracted from the individual stations, the altitudinal amplification trend was evaluated by regressing *Q*_ALT_ against *ALT* to obtain the amplification factor (*Q*_ALTAMP_, in °C km^-1^50-yrs^-1^) over the period 1961–2010.

Besides, if assuming that the temperature change in a high-elevation region is predominately controlled by altitude and latitude, then the *EC*_ALT_, and *EC*_LAT_ will be estimated using the model of fit *y* = b_1_ *x*_1_ + b_2_ *x*_2_ + *c*, where the long-term average T_MEAN_ (T_MIN_ or T_MAX_; in °C), and the *ALT* and *LAT* at individual stations within the region will be used as the dependent (*y*) and independent variables (*x*_1_ and *x*_2_), respectively. The negative values of b_1_ and b_2_ will be taken as the *EC*_ALT_ and *EC*_LAT_, respectively. The extraction of *Q*_ALT_ from *Q*_TOTAL_ for each station is conducted either using eq (4), where the *EC*_LONG_ is assumed to be zero, or using the following equation:

$$Q_{ALT}=\frac{Q_{TOTAL}\times EC_{ALT}\times ALT}{\sqrt{{\left(EC_{ALT}\times ALT\right)}^{2}+{\left(EC_{LAT}\times LAT\right)}^{2}}}.$$ (5)

Furthermore, if assuming that the temperature change in a high-elevation region is only controlled by altitude (both *EC*_LAT_ and *EC*_LONG_ are considered to be zero), *Q*_ALT_ will be equal to *Q*_TOTAL_,

$$Q_{ALT}=\frac{Q_{TOTAL}\times EC_{ALT}\times ALT}{\sqrt{{\left(EC_{ALT}\times ALT\right)}^{2}}}=\frac{Q_{TOTAL}\times EC_{ALT}\times ALT}{EC_{ALT}\times ALT}=Q_{TOTAL}.$$ (6)

It is clear that this equation is a special case of eq (4) or eq (5).

#### 2.2.3 Test of regional amplification

The regional amplification was tested using the paired-region comparison method [19]. Each of the paired high and lower elevation regions were selected using a method similar to the belt transect method. Each paired regions are located at the same latitudes, and has the equal longitude range. The sampled area is a northeast-southwest parallelogram for the Appalachian Mountains, and its west low-lying counterpart (Table 1).

**Table 1:** The latitudes, longitudes, and average altitudes of the paired regions across the globe.

No.	Paired regions	Latitude	Longitude	Avg alt (km)	n
Annual mean minimum temperature					
1a	North Tibetan Plateau	34–38°N	93–102°E	3.1365	23
1b	Loess Plateau	34–38°N	103–112°E	1.0173	112
2a	East Loess Plateau	34–38°N	107–112°E	0.8036	83
2b	North China Plain	34–38°N	114–119°E	0.0782	17
3a	Alps	45–48.5°N	5.5–16.5°E	0.9598	58
3b	East lower region	45–48.5°N	17°E–28°E	0.2681	17
4a	Southeast Rockies (USA)	36–41°N	104–109°W	2.0804	17
4b	East lower region	36–41°N	86–91°W	0.1793	53
5a	Appalachian Mountains	35–46°N	68.5–72.6°W at 46°N 81.7–85.8°W at 35°N	0.4757	56
5b	West lower region	35–46°N	73–77.1°W at 46°N 86.2–90.3°W at 35°N	0.2593	66
Annual mean maximum temperature					
1a	North Tibetan Plateau	34–38°N	93–102°E	3.1365	23
1b	Loess Plateau	34–38°N	103–112°E	1.0452	118
2a	East Loess Plateau	34–38°N	107–112°E	0.8044	85
2b	North China Plain	34–38°N	114–119°E	0.0782	17
3a	Alps	45–48.5°N	5.5–16.5°E	0.9905	53
3b	East lower region	45–48.5°N	17°E–28°E	0.2681	17
4a	Southeast Rockies (USA)	36–41°N	104–109°W	2.0747	17
4b	East lower region	36–41°N	86–91°W	0.1764	54
5a	Appalachian Mountains	35–46°N	68.5–72.6°W at 46°N 81.7–85.8°W at 35°N	0.4759	54
5b	West lower region	35–46°N	73–77.1°W at 46°N 86.2–90.3°W at 35°N	0.2546	66

However, the regional amplification was not only evaluated for T_MIN_ and T_MAX_ as for T_MEAN_ [19], but also for T_MIN_ and T_MAX_ as a whole. (1) The regional amplification was evaluated for T_MIN_ and T_MAX_ separately when a similar asymmetric warming in T_MIN_ and T_MAX_ occurs between one paired regions; that is, the magnitude of the trend is greater for T_MIN_ than T_MAX_ (or for T_MAX_ than T_MIN_) in both high- and low-elevation regions. (2) The regional amplification was evaluated for T_MIN_ and T_MAX_ as a whole (the average magnitude of T_MIN_ and T_MAX_ trends was used for comparison) when an opposite asymmetric warming in T_MIN_ and T_MAX_ occurs between one paired regions; that is, the magnitude of the trend is greater for T_MIN_ than T_MAX_ in one region, whereas the magnitude of the trend is greater for T_MAX_ than T_MIN_ in its counterpart. In this situation, the separate analysis of regional amplification for T_MIN_ and T_MAX_ would not only make the difference in T_MIN_ or T_MAX_ (or both) appear very large between the paired regions, but would also make the warming in T_MIN_ or T_MAX_ look even weaker at times for the high-elevation region than its lower counterpart, and vice versa, even if the average warming (the magnitude of the T_MEAN_ trend) is greater in the high-elevation region than its lower counterpart.

## Results

### 3.1 Altitudinal amplification

**Fig 3:**
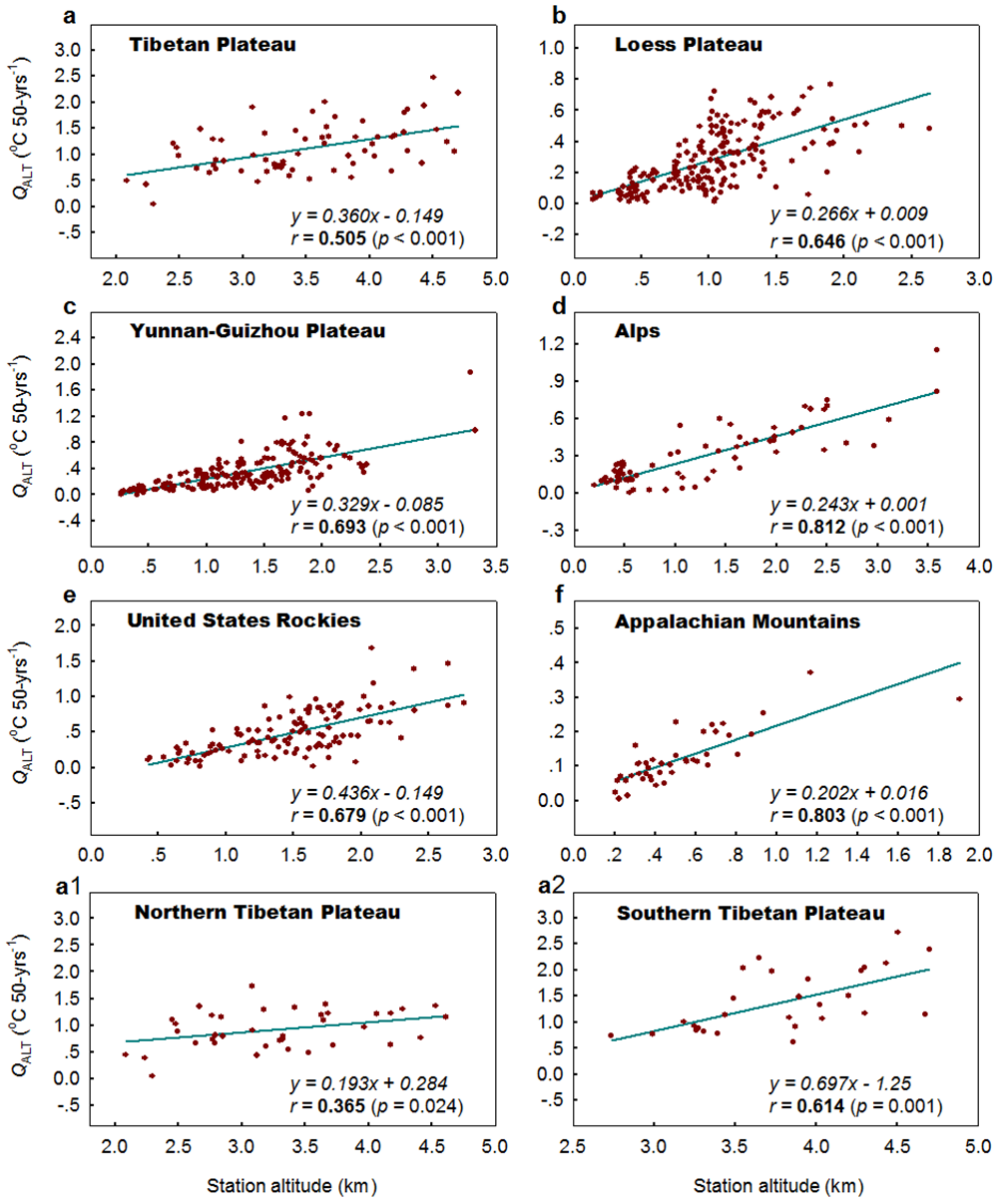
Relationship between altitudinal warming components (*Q*_ALT_s) of annual mean minimum temperature and station altitudes for the high-elevation regions across the globe. Dots represent *Q*_ALT_s, and dark cyan lines indicate linear regression lines. The magnitude of altitudinal amplification trend (*Q*_ALTAMP_, the gradient of the regression line) is expressed in °C km^-1^50-yrs^-1^. Pearson correlation coefficients (*r*) are shown with two-tailed *p* values in parentheses. Significant coefficients (at the 0.05 level) are set in bold.

**Fig 4:**
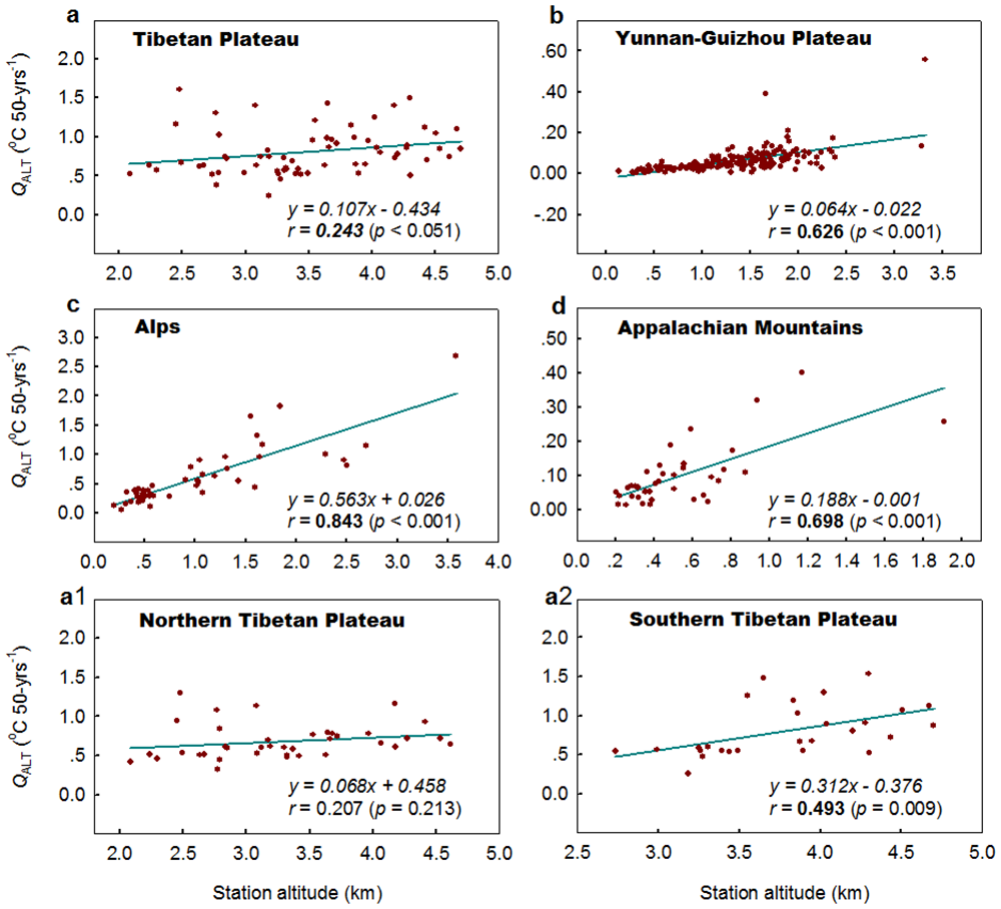
Same as Fig 3 but for annual mean maximum temperature, with the marginally significant coefficient (at the 0.10 level) in italic bold.

Figs 3 and 4 depict the relationship between the altitudinal warming components (*Q*_ALT_s) and station altitudes within each high-elevation region for T_MIN_ and T_MAX_, respectively. A significant altitude amplification trend in T_MIN_ is detected in all the high-elevation regions tested (the Tibetan Plateau, Loess Plateau, Yunnan-Guizhou Plateau, Alps, US Rocky Mountains, and Appalachian Mountains), whereas a significant or a marginally significant altitude amplification trend in T_MAX_ is observed in four of the high-elevation regions (significant: the Yunnan-Guizhou Plateau, Alps, and Appalachian Mountains; marginally significant: the Tibetan Plateau). No altitudinal amplification in T_MAX_ is detected in the Loess Plateau, and US Rocky Mountains.

In the regions where altitudinal amplification trends have been confirmed for T_MIN_ and T_MAX_, the magnitudes of the amplification trends are generally greater for T_MIN_ than for T_MAX_ (Table 2). If the magnitude of the altitudinal amplification trend is taken as zero when no altitudinal amplification trend is detected, the average magnitude of altitudinal amplification trends for the six regions is 0.306 ± 0.086 °C km^-1^ 50-yrs^-1^ and 0.154 ± 0.213 °C km^-1^ 50-yrs^-1^ for T_MIN_ and T_MAX_, respectively. This indicates a remarkable asymmetry in the altitudinal amplification between T_MIN_ and T_MAX_. The average magnitude of amplification trends is 0.33 times greater for T_MIN_ but 0.33 times smaller for T_MAX_ compared with the magnitude for T_MEAN_ (0.230 ± 0.073 °C km^-1^) during the period 1961–2010.

**Table 2:** Summary of the regional warming trend [*Q*_REG_(°C 50-yrs^-1^)], effect coefficients of altitude, latitude and longitude [*EC*_ALT_, *EC*_LAT_, and *EC*_LONG_ (°C km^-1^)], and altitudinal amplification trend [*Q*_ALTAMP_ (°C km^-1^50-yrs^-1^)] for the high-elevation regions across the globe.

No.	Region	*Q*_REG_	*EC*_ALT_	*EC*_LAT_	*EC*_LONG_	*Q*_ALTAMP_	n
Annual mean temperature							
1	Tibetan Plateau	**1.867**	6.0596	0.0091	0.0013	**0.271**	66
2	Loess Plateau	**1.595**	4.8945	0.0045	0.0017	**0.353**	196
3	Yunnan-Guizhou Plateau	**0.779**	3.9007	0.0067	0.0024	**0.148**	183
4	Alps	**1.639**	5.4186	0.0089	0	**0.212**	70
5	United States Rockies	**1.321**	5.7474	0.0060	0	**0.213**	117
6	Appalachian Mountains	**1.754**	5.7996	0.0092	0	**0.180**	42
1a	Northern Tibetan Plateau	**1.955**	6.5443	0.0104	0.0028	**0.176**	38
1b	Southern Tibetan Plateau	**1.847**	5.5027	0.0089	0	**0.522**	28
Annual mean minimum temperature							
1	Tibetan Plateau	**2.395**	5.7519	0.0101	0.0009	**0.360**	66
2	Loess Plateau	**1.939**	4.9744	0.0068	0.0025	**0.266**	194
3	Yunnan-Guizhou Plateau	**1.251**	4.1371	0.0064	0.0009	**0.329**	180
4	Alps	**1.499**	5.7559	0.0077	0	**0.243**	55
5	United States Rockies	**1.263**	6.6778	0.0055	0	**0.436**	113
6	Appalachian Mountains	**1.945**	4.8559	0.0080	0	**0.202**	41
1a	Northern Tibetan Plateau	**2.420**	6.5421	0.0120	0.0023	**0.193**	38
1b	Southern Tibetan Plateau	**2.495**	5.7618	0.0089	0	**0.697**	28
Annual mean maximum temperature							
1	Tibetan Plateau	**1.582**	6.1228	0.0089	0.0013	***0*.*107***	65
2	Loess Plateau	**1.606**	0	0	0	0	196
3	Yunnan-Guizhou Plateau	**0.644**	4.0497	0.0073	0.0059	**0.064**	181
4	Alps	**1.513**	6.2238	0.0036	0	**0.563**	51
5	United States Rockies	**1.547**	0	0	0	0	113
6	Appalachian Mountains	**1.550**	7.1067	0.0100	0	**0.188**	38
1a	Northern Tibetan Plateau	**1.779**	6.5600	0.0099	0.0036	0.068	38
1b	Southern Tibetan Plateau	**1.324**	6.0120	0.0089	0	**0.312**	27

The results for annual mean temperature are cited from Wang et al.[19] with a correction of *Q*_ALTAMP_ for the US Rockies. Significant trends (at the 0.05 level) are set in bold, with the marginally significant trend (at the 0.10 level) in italic bold.

Similar results are obtained (Table 3) provided that temperature change in high-elevation regions is predominately controlled by two variables (altitude and latitude) rather than three variables (altitude, latitude and longitude). The average magnitude of the altitudinal amplification trends for the six regions is0.300 ±0.089 °C km^-1^50-yrs^-1^ and 0.155±0.212 °C km^-1^ 50-yrs^-1^for T_MIN_ and T_MAX_, respectively;0.32 times greater for T_MIN_ but 0.32 times smaller for T_MAX_ compared with that (0.228 ±0.069 °C km^-1^) for T_MEAN_ during the same period. This indicates that the longitude effect, though significant in three of the regions tested, is almost negligible in quantifying the altitudinal amplification trends in high-elevation regions.

**Table 3:** Same as Table 2 but provided that the temperature change is predominately controlled by two variables (altitude and latitude) rather than three variables (altitude, latitude, and longitude).

No.	Region	*EC*_ALT_	*EC*_LAT_	*Q*_ALTAMP_	n
Annual mean temperature					
1	Tibetan Plateau	5.6761	0.0095	**0.271**	66
2	Loess Plateau	4.2662	0.0054	**0.341**	196
3	Yunnan-Guizhou Plateau	3.2008	0.0087	**0.148**	183
4	Alps	5.4186	0.0089	**0.212**	70
5	United States Rockies	5.7474	0.0060	**0.213**	117
6	Appalachian Mountains	5.7996	0.0092	**0.180**	42
1a	Northern Tibetan Plateau	5.7606	0.0093	**0.192**	38
1b	Southern Tibetan Plateau	5.5027	0.0089	**0.522**	28
Annual mean minimum temperature					
1	Tibetan Plateau	5.7519	0.0101	**0.366**	66
2	Loess Plateau	4.0506	0.0080	**0.239**	194
3	Yunnan-Guizhou Plateau	3.8730	0.0071	**0.313**	180
4	Alps	5.7559	0.0077	**0.243**	55
5	United States Rockies	6.6778	0.0055	**0.436**	113
6	Appalachian Mountains	4.8559	0.0080	**0.202**	41
1a	Northern Tibetan Plateau	5.9046	0.0112	**0.202**	38
1b	Southern Tibetan Plateau	5.7618	0.0089	**0.697**	28
Annual mean maximum temperature					
1	Tibetan Plateau	5.7493	0.0094	**0.112**	65
2	Loess Plateau	0	0	0	196
3	Yunnan-Guizhou Plateau	2.4075	0.0122	**0.067**	181
4	Alps	6.2238	0.0036	**0.563**	51
5	United States Rockies	0	0	0	113
6	Appalachian Mountains	7.1067	0.0100	**0.188**	38
1a	Northern Tibetan Plateau	5.5450	0.0086	0.072	38
1b	Southern Tibetan Plateau	6.0120	0.0089	**0.312**	27

However, if assuming that temperature change in high-elevation regions is only controlled by altitude, contrasting results are obtained for T_MEAN_[19], T_MIN_ and T_MAX_(Table 4). The differing results for T_MEAN_, T_MIN_ and T_MAX_ among the high-elevation regions could be attributed to the difference in signal intensity of the *Q*_TOTAL_ values[28] and the region-specific interactions between altitude and latitude [19]in the individual regions (see the ‘Discussion‘section for details).

**Table 4:** Relationships between station warming rates (*Q*_TOTAL_, °C 50-yrs^-1^) and station altitudes (km) in the high-elevation regions across the globe.

No.	Region	Simple linear regression				n
		*r*	*p*	*Q*_TOTALAMP_	*B*	
Annual mean temperature						
1	Tibetan Plateau	0.027	= 0.830	0.027	1.761	66
2	Loess Plateau	0.280	<0.001	**0.394**	1.175	196
3	Yunnan-Guizhou Plateau	0.287	<0.001	**0.224**	0.502	183
4	Alps	-0.119	= 0.327	-0.062	2.096	70
5	United States Rockies	0.287	= 0.002	**0.229**	0.404	117
6	Appalachian Mountains	0.051	= 0.746	0.070	1.327	42
1a	Northern Tibetan Plateau	-0.079	= 0.635	-0.087	2.252	38
1b	Southern Tibetan Plateau	0.617	<0.001	**0.610**	-0.613	28
Annual mean minimum temperature						
1	Tibetan Plateau	0.066	= 0.601	0.091	2.090	66
2	Loess Plateau	0.082	= 0.256	0.189	1.840	194
3	Yunnan-Guizhou Plateau	0.299	<0.001	**0.339**	0.837	180
4	Alps	-0.124	= 0.368	-0.163	1.987	55
5	United States Rockies	0.282	= 0.002	**0.344**	0.797	113
6	Appalachian Mountains	-0.027	= 0.865	-0.066	1.734	41
1a	Northern Tibetan Plateau	-0.105	= 0.532	-0.148	2.924	38
1b	Southern Tibetan Plateau	0.468	= 0.012	**0.783**	-0.606	28
Annual mean maximum temperature						
1	Tibetan Plateau	-0.262	= 0.035	**-0.252**	2.487	65
2	Loess Plateau	0.032	= 0.656	0.047	1.651	196
3	Yunnan-Guizhou Plateau	0.044	= 0.554	-0.085	0.931	181
4	Alps	0.179	= 0.210	0.169	1.755	51
5	United States Rockies	0.108	= 0.256	0.171	1.308	113
6	Appalachian Mountains	0.062	= 0.712	0.120	1.070	38
1a	Northern Tibetan Plateau	-0.334	= 0.040	**-0.332**	2.887	38
1b	Southern Tibetan Plateau	0.331	= 0.091	***0*.*306***	0.172	27

*Q*_TOTALAMP_ denotes altitudinal amplification trend of *Q*_TOTAL_s, expressed in °C km^-1^50-yrs^-1^. *B* represents intercept. Pearson correlation coefficient (*r*) is given with the two-tailed *p* value for each case. Significant trends (at the 0.05 level) are set in bold, with the marginally significant ones (at the 0.10 level) in italic bold. The results for annual mean temperature are cited from Wang et al. [19].

### 3.2 Regional amplification

**Fig 5:**
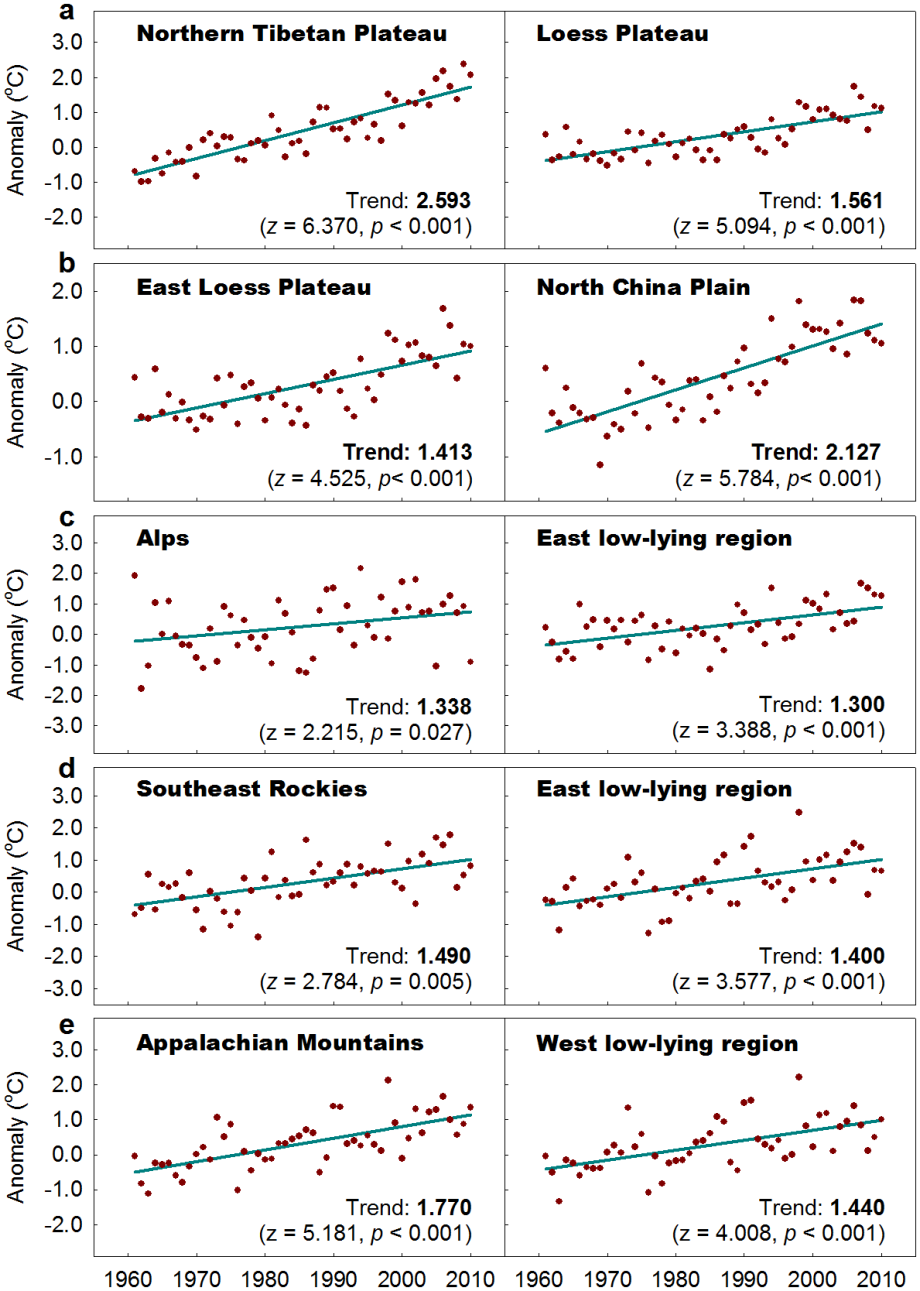
Monotonic trends of annual mean minimum temperature during 1961–2010 over the paired regions shown across the globe. The regional anomaly values are produced by simple averaging of the individual station anomaly values (relative to the 1961 to 1990 means) within each region. The trend is extracted using the Mann-Kendall test method, and expressed in °C 50-yrs^-1^, with the statistic *z* and its *p* value in parentheses. The significant trend (at the 0.05 level) is set in bold.

**Fig 6:**
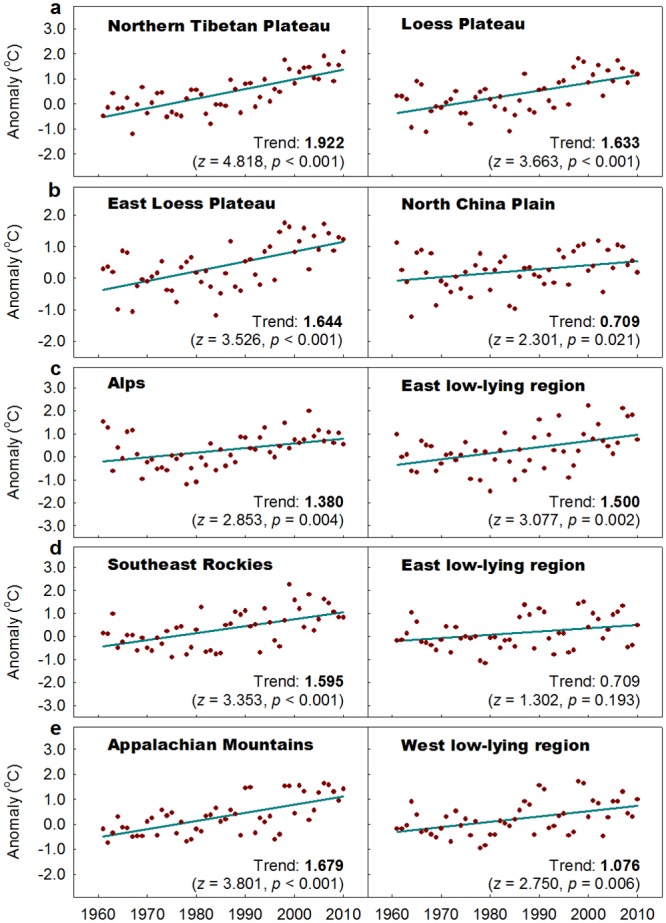
Same as Fig 5 but for annual mean maximum temperature.

Figs 5 and 6 depict the monotonic trends (1961–2010) in the paired regions for T_MIN_ and T_MAX_, respectively. Among the five paired regions, the magnitude of the T_MAX_ trend is larger than the T_MIN_ trend for the Alps and its low-lying counterpart, whereas the magnitude of the T_MIN_ trend is larger than the T_MAX_ trend for the Appalachian Mountains and its low-lying counterpart. Despite this difference between these two paired regions, similar asymmetric warming is detected in each paired regions. It is clear that greater warming is only observed in T_MIN_ for the Alps than its east low-lying counterpart, whereas greater warming is detected in both T_MIN_ and T_MAX_ in the Appalachian Mountains than in its low-lying counterpart.

For other three paired regions, opposite asymmetric warming is observed between each paired regions. The magnitude of the T_MIN_ trend is larger than the T_MAX_ trend on the Northern Tibetan Plateau, whereas the magnitude of the T_MIN_ trend is smaller than that of the T_MAX_ trend in its eastern lower-elevation counterpart (the sampled Loess Plateau). The magnitude of the T_MIN_ trend is smaller than that of the T_MAX_ trend in the East Loess Plateau and the Southeast Rocky Mountains, whereas the T_MIN_ trend is larger than the T_MAX_ trend in their low-lying counterparts (the North China Plain and the eastern low-lying region, respectively). When the regional amplification is estimated for T_MIN_ and T_MAX_ as a whole, the warming is greater on the North Tibetan Plateau than the sampled Loess Plateau (2.26 versus 1.60 °C)and in the Southeast Rocky Mountains than its eastern low-lying region (1.54 versus 1.05 °C) during 1961–2010. Greater warming also occurred in the East Loess Plateau than on the North China Plain (1.53 versus 1.42 °C) during the same period.

## Discussion

Many factors might have played a role in shaping the patterns of temperature change in high-elevation regions across the globe. However, it is worth noting that these factors might have distinct effects in terms of their magnitudes and directions, and can be defined as basic and non-basic factors in terms of their relative status in the complex interaction hierarchy.

Barry [2] stated that climate in mountain regions is controlled by four basic factors: altitude, latitude, continentality, and topography. A previous study has shown that altitude and latitude are major factors in determining the geographical pattern of temperature change in the Alps [12]. Notably, the effect of energy balance variation on surface temperature was found to be amplified with decreasing temperature in the environment as a result of the functional shape of the Stefan-Boltzmann law [29–30]. Provided that this energy balance effect is a fundamental theoretical basis on warming amplification under lower temperature rather than a partial explanation of larger temperature trends in polar and high-altitude climate [29–30], because the higher the altitude (latitude), the lower the temperature [2], the magnitudes of station temperature trends across a high-elevation region will be closely related to both *ALT* and *LAT*. Furthermore, provided that there is a clear temperature gradient with longitude due to continentality, then this will remain true for *LONG* as well. It is based on these observations and deductions that Wang *et al*.[19] have developed the altitudinal warming component extraction equation (AWCE equation).

In fact, a significant negative relationship between *T*_*AVG*_ and *ALT*, *LAT* and *LONG* (between *T*_*AVG*_ and *ALT* and *LAT*) was detected for three (five) of the eight high-elevation regions tested [19]. A significant altitudinal amplification trend in T_MEAN_ was detected in each region by extracting Q_ALT_ from Q_TOTAL_ at the individual stations in each region using the AWCE equation [19]. Because the same holds good for T_MIN_ and T_MAX_ for the regions where a significant negative relationship exists between *T*_*AVG*_ and *ALT*, *LAT* and *LONG* or between *T*_*AVG*_ and *ALT* and *LAT*, significant altitudinal amplification trends are detected for T_MIN_ and T_MAX_ in four regions, consistent with the T_MEAN_ results from these regions. In the Loess Plateau and the US Rocky Mountain regions, significant altitudinal amplification trends are detected for T_MIN_, whereas no altitudinal amplification trends are observed for T_MAX_ because no *EC*_ALT_, *EC*_LAT_ and *EC*_LONG_ are estimated for these two regions due to the small difference in daytime temperatures among the individual stations across both regions.

Quantitatively, the asymmetric altitudinal amplification trends in T_MIN_ and T_MAX_ are consistent with the regional temperature trends in T_MIN_ and T_MAX_ for all the regions, with the exception of the US Rocky Mountains (Table 2). The elevation-dependent warming in minimum temperatures on and around the Tibetan Plateau has been demonstrated by plotting the trends at individual elevation bands versus elevation in the previous studies [11, 14]. In contrast, You et al. [13] failed to substantiate altitudinal amplification trends in most temperature extreme indices derived from daily minimum and maximum temperatures in the eastern and central Tibetan Plateau.

The asymmetric changes in T_MIN_ and T_MAX_ have been reported for a number of large regional series [31–32] and for certain high-elevation regions [15, 33–36]. A stronger warming for T_MIN_ than T_MAX_ has been observed in the Tibetan Plateau[35–36], the eastern Loess Plateau [37] and the stations located in different elevation ranges in the latitudinal bands 30°N-70°N [34]. Greater warming has been observed in T_MIN_ than T_MAX_ in the Swiss Alps over the period1901–1992[33], whereas similar changes in the minimum and maximum temperatures have been detected in the mountainous regions of Central Europe over the period1901–1990[17]. The greater warming for T_MAX_ than T_MIN_ observed in this study indicates a shift from stronger warming in T_MIN_ to stronger warming in T_MAX_ for the Alps during 1961–2010. Moreover, McGuire *et al*. [15] found significant warming in T_MAX_ but not in T_MIN_ in the Rocky Mountain Front Range during 1953–2008. The presence of a cooling signal in T_MIN_ along the Front Range [15] and the weaker warming for T_MIN_ than T_MAX_ for the entire US Rocky Mountain region observed in the current study are likely related to changes in regional and local land-use practices [15] and possible changes in atmospheric circulation [38].

In terms of the longitude effect, it is difficult to interpret the meaning of *EC*_LONG_ except for the above deduction. Nevertheless, similar results are observed for T_MEAN_, T_MIN_ and T_MAX_ in the high-elevation regions tested (Table 3) when two variables (altitude and latitude) are considered instead of three variables (altitude, latitude and longitude). This indicates that the altitudinal amplification trend in a high-elevation region can be well approximated when these two basic variables are taken into account.

Why is Q_ALT_ so different from Q_TOTAL_ when they are each regressed against *ALT*? First, this is due to Q_TOTAL_s being predominately controlled by both altitude and latitude over a high-elevation region, while Q_ALT_s are associated only with altitude, as the name suggests. Consequently, the correlation between Q_TOTAL_ and *ALT* can be reduced by a huge amount of noise, whereas no noise affects the correlation between Q_ALT_ and *ALT*. Relative to the effect (signal) of the target independent variable (*ALT*), the effect of the non-target independent variable (*LAT*), as well as the interacting effect of *LAT* and *ALT*, should be considered noise in the statistical analysis. Second, the magnitude of the noise impact on the correlation between Q_TOTAL_ and *ALT* varies from region to region, depending on the signal intensity (SI) of Q_TOTAL_ in individual regions. According to the SI concept [28], the Q_TOTAL_SI (signal quantity per unit area) in a region is approximately proportional to the number of available stations in a region and is inversely proportional to the total area of the region. It is probably due to the very high Q_TOTAL_SI for the Loess Plateau, the Yunnan-Guizhou Plateau, and the US Rockies, but very low Q_TOTAL_ SI for the Tibetan Plateau and the Appalachian Mountains, that a significant correlation between Q_TOTAL_ and *ALT* is detected for the former, but not for the latter in terms of T_MEAN_ (Table 4). The same holds true for T_MIN_, excepting that a positive but non-significant correlation between Q_TOTAL_ and *ALT* is observed for the Loess Plateau (Table 4).

Furthermore, the contrasting correlations between Q_TOTAL_ and *ALT* among these regions (particularly between the two sub-regions of the Tibetan Plateau) could be partly attributable to the effect of topography (the underlying feature of the available stations)on them. As revealed in a previous study [19], the underlying topography of the available stations in the Northern Tibetan Plateau (NTP) is characterized by a significant negative ***s***patial ***co***rrelation between the station ***al***titudes and station ***la***titudes (*SCOALLA*), whereas no significant negative *SCOALLA* occurs in the Southern Tibetan Plateau (STP); indicating that the altitude effect could be cancelled out (or overwhelmed) by the latitude effect in the NTP while not in the STP [19]. Therefore, the correlation between Q_TOTAL_ and *ALT* is non-significant (negatively significant) for T_MEAN_ and T_MIN_ (T_MAX_) over the NTP, while the correlation between Q_TOTAL_ and *ALT* is positively significant (marginally significant) for T_MEAN_ and T_MIN_ (T_MAX_) over the STP (Table 4). Owing to that, the correlation between Q_TOTAL_ and *ALT* for the entire Tibetan Plateau is a reflection of those two sub-regions, and the number of stations from the NTP is obviously larger than that from the STP, the correlation between Q_TOTAL_ and *ALT* for the entire Tibetan Plateau is closer to that of the NTP rather than the STP (Table 4).

In addition, it should be noted that although the negative *SCOALLA* has no direct influence on the correlation between Q_ALT_ and *ALT*, it may more or less affect the long-term average values of T_MEAN_, T_MIN_ and T_MAX_, and consequently the *EC*_ALT_ and *EC*_LAT_. This, in turn, could have affected the magnitudes of Q_ALT_s, and therefore the relationship between Q_ALT_ and *ALT*. It is probably due to this indirect influence that the magnitudes of altitudinal amplification trends appear underestimated for the Tibetan Plateau and the Northern Tibetan Plateau relative to the Southern Tibetan Plateau (Table 2). However, the topographical effect is difficult to quantify, and this effect can only be taken as noise relative to the direct effects of altitude and latitude. Hence it is not taken into account in the quantitative estimation of *Q*_ALT_s.

For comparative purpose, the global base *EC*_ALT_ and *EC*_LAT_ were also estimated for T_MIN_ and T_MAX_. The base *EC*_ALT_ and *EC*_LAT_ were computed according to the data from all the high (≥200 m above sea level) and low (<200 m above sea level) elevation stations, respectively, for either index using the same method as for T_MEAN_ [19]. The result shows that the global base *EC*_ALT_ values in T_MEAN_, T_MIN_ and T_MAX_(4.9±0.9, 5.0±1.2and 3.1±2.8 °C km^-1^, respectively) are smaller than the average *EC*_ALT_ values in T_MEAN_, T_MIN_ and T_MAX_ (5.3±0.8, 5.4±0.9 and 3.9±3.2 °C km^-1^, respectively) for the six high-elevation regions, and the global base *EC*_LAT_ values in T_MEAN_, T_MIN_ and T_MAX_(0.0049±0.0037, 0.0045 ±0.0028, and 0.0048 ±0.0036 °C km^-1^, respectively) are clearly smaller than the average *EC*_LAT_ values in T_MEAN_, T_MIN_ and T_MAX_ (0.0074±0.0020, 0.0074±0.0016, and 0.0050±0.0044 °Ckm^-1^, respectively) for these regions. This suggests that there exists not only an enhanced *EC*_ALT_ but also an enhanced *EC*_LAT_ in the high-elevation regions. Therefore, a greater warming usually occurs in high-elevation regions relative to their lower elevation counterparts.

The slightly greater warming in T_MIN_ and even weaker warming in T_MAX_ in the Alps relative to its low-lying counterpart are likely associated with the greater urban heat effect in the low-elevation area because the urban heat effect is generally greater at low-elevation sites [9, 17]. On the other hand, the unusually stronger warming in T_MIN_ relative to T_MAX_ over the North China Plain could have partially resulted from the unusually large urban heat effect on T_MIN_ relative to T_MAX_. The urban heat effect is primarily a nocturnal phenomenon in certain places around the world [39–40]. The North China Plain may be one such place. The large difference between changes in T_MIN_ and T_MAX_ between the East Loess Plateau and the North China Plain is also likely related to the barrier effect of the Taihang Mountains, which run from north to south in North China, forming a natural boundary between the paired regions and a physical barrier to the southeast summer monsoon in China. In fact, the trends in various precipitation indices also differ between the East Loess Plateau and the North China Plain due to this barrier effect. For instance, Fan *et al*. [37] observed a significant decreasing trend (-15.05mm 50-yrs^-1^) in annual total precipitation on wet days (PRCPTOT) over the entire Shanxi Province, whereas a non-significant trend was observed in PRCPTOT over the northern half of the North China Plain. These two regions are nearly equivalent to the East Loess Plateau and North China Plain in this study.

Considerable seasonal variations in trend magnitude have been observed [11, 14, 30, 38]. The warming amplifications in high-elevation regions may vary greatly on sub-annual scales due to changes in atmospheric circulation and local processes, such as snow albedo and water vapor feedbacks [38, 41]. An improved understanding of altitudinal amplification on sub-annual scales may have more important bearings on societal, ecological and physical systems in high-elevation regions. Therefore it is of great important to characterize the seasonal and monthly pictures of warming amplification of T_MEAN_, T_MIN_ and T_MAX_ on a global scale.

## Conclusions

In this study, analysis of T_MIN_ and T_MAX_ series (1961–2010) show a significant altitudinal amplification trend in T_MIN_ (T_MAX_) in six (four) of the high-elevation regions tested. The average magnitude of altitudinal amplification trends for the six high-elevation regions is substantially larger (smaller) for T_MIN_ (T_MAX_) [0.306±0.086 °C km^-1^(0.154±0.213 °C km^-1^)] than T_MEAN_ (0.230±0.073 °C km^-1^) in the period 1961–2010. Similar results are obtained when the effects of two variables (altitude and latitude) are considered instead of three variables (altitude, latitude and longitude). For the five paired high- and low-elevation regions available, regional amplification is detected in four high-elevation regions for T_MIN_ and T_MAX_ (respectively or as a whole), whereas it is only observed for T_MIN_ in the fifth high-elevation region. Qualitatively, highly (largely) consistent results are observed for T_MIN_ (T_MAX_) compared with those for T_MEAN_. The results for T_MIN_ (T_MAX_) are basically in conformity with our expectations. These results confirm the effectiveness of the AWCE equation in quantifying altitudinal amplification trend within a high-elevation region. Future study is required to explore the seasonal and monthly patterns of warming amplification trends in T_MEAN_, T_MIN_ and T_MAX_ on a global scale.
